# Antenna-assisted picosecond control of nanoscale phase transition in vanadium dioxide

**DOI:** 10.1038/lsa.2016.173

**Published:** 2016-10-21

**Authors:** Otto L Muskens, Luca Bergamini, Yudong Wang, Jeffrey M Gaskell, Nerea Zabala, CH de Groot, David W Sheel, Javier Aizpurua

**Affiliations:** 1 Physics and Astronomy, Faculty of Physical Sciences and Engineering, University of Southampton, Southampton SO17 1BJ, UK; 2 Department of Electricity and Electronics, FCT-ZTF, UPV-EHU, Bilbao 48080, Spain; 3 Materials Physics Center, CSIC-UPV/EHU and DIPC, San Sebastian 20018, Spain; 4 Nano Group, Faculty of Physical Sciences and Engineering, University of Southampton, Southampton SO17 1BJ, UK; 5 Materials and Physics Research Centre, University of Salford, Manchester M5 4WT, UK

**Keywords:** insulator-metal phase transition, nanoantenna, plasmonics, VO_2_

## Abstract

Nanoscale devices in which the interaction with light can be configured using external control signals hold great interest for next-generation optoelectronic circuits. Materials exhibiting a structural or electronic phase transition offer a large modulation contrast with multi-level optical switching and memory functionalities. In addition, plasmonic nanoantennas can provide an efficient enhancement mechanism for both the optically induced excitation and the readout of materials strategically positioned in their local environment. Here, we demonstrate picosecond all-optical switching of the local phase transition in plasmonic antenna-vanadium dioxide (VO_2_) hybrids, exploiting strong resonant field enhancement and selective optical pumping in plasmonic hotspots. Polarization- and wavelength-dependent pump–probe spectroscopy of multifrequency crossed antenna arrays shows that nanoscale optical switching in plasmonic hotspots does not affect neighboring antennas placed within 100 nm of the excited antennas. The antenna-assisted pumping mechanism is confirmed by numerical model calculations of the resonant, antenna-mediated local heating on a picosecond time scale. The hybrid, nanoscale excitation mechanism results in 20 times reduced switching energies and 5 times faster recovery times than a VO_2_ film without antennas, enabling fully reversible switching at over two million cycles per second and at local switching energies in the picojoule range. The hybrid solution of antennas and VO_2_ provides a conceptual framework to merge the field localization and phase-transition response, enabling precise, nanoscale optical memory functionalities.

## Introduction

With the miniaturization of optical components and the convergence of electronic and photonic technologies, ultracompact devices are needed to control and switch light on length scales comparable to the optical wavelength. Resonant plasmonic antennas at visible and near-infrared wavelengths offer the capability to concentrate energy in space and time and enhance light–matter interaction and nonlinear response^1, 2^. To achieve order-unity switching effects, hybrid devices combining plasmonic antennas with materials showing a structural or electronic phase transition provide unique opportunities because they can provide very large changes in optical response. Recent investigations of such hybrid systems include switchable metal hydrides^3, 4, 5^, interface-mediated solid-liquid transitions of gallium^6^, resonant bonding phase-change materials^7, 8, 9, 10^ and insulator-to-metal transitions (IMTs) in correlated oxides^11^. Chalcogenide phase-change materials, such as Ge:Sb:Te (GST), allow for non-volatile writing and rewriting of photonic devices using a pulsed laser^8, 9, 10, 12, 13^. Excitation of this phase-change response using the optical near-field opens up routes for achieving ultrasmall switching volumes and low-energy devices^14, 15^.

Compared with chalcogenide phase-change materials, which provide rewritable, non-volatile memory functionality at relatively high temperatures, vanadium dioxide (VO_2_) is characterized by a reversible IMTs at only modestly elevated temperatures of ~68 °C^11, 16^. This phase transition can be driven using ultrafast laser pulses, where the precise mechanisms depend on the pulse duration and energy injection mechanisms involved, with a structural bottleneck as short as 80 fs^17, 18, 19^. While strong nonequilibrium physics occur on ultrafast time scales, the VO_2_ IMT is quasi-thermal for times >1 ps^18^.

Several studies have reported the effects of a global, thermally or optically driven phase transition in VO_2_ on the plasmonic response of nanoparticles and metamaterials over a broad spectral range from terahertz^20, 21, 22, 23^, mid-infrared^24, 25^, near-infrared^26, 27, 28^ to visible^29, 30^. In the THz range, the giant electric-field strength in micrometer-sized antenna feed gaps has been used to overcome the Coulomb potential barrier for IMT, analogous to DC field-induced switching^21^. In the visible and near-infrared, studies of plasmonic effects on the optical excitation of the IMT have focused on hot-electron injection-driven ultrafast processes^31, 32^ and on non-resonant, continuous-wave-mediated plasmonic heating^28, 33^.

Here, we use picosecond pulsed excitation to drive a highly localized phase-transition around resonant plasmonic nanoantennas. The picosecond pulsed regime is of relevance for many practical applications, and nonlinear devices on this time scale are needed to enable all-optical switching^34^. Our experiments are enabled by very high-quality VO_2_ films that are grown with a surface roughness <10 nm using atmospheric chemical vapor deposition^35^. Gold nanoantennas were fabricated on top of these films, forming a hybrid system together with the VO_2_, with an effective response that is governed by the nanoscale interplay between the plasmonic and IMT components. Resonant antenna-assisted switching is studied using optical pump–probe experiments, where we perform picosecond pulsed excitation at a fixed pump wavelength while tuning the antenna resonance condition via the antenna length. Multifrequency arrays of crossed antennas^36^ were specifically designed to individually address the cross-coupling of optically induced phase transitions between adjacent antennas with mutual separations <100 nm. Antenna-assisted optical pumping is identified through the dependence of the IMT switching on the polarizations of the pump and readout, on the pulse energy of the optical pump, and on the cycling rate and hysteresis of the IMT. A model combining resonant antenna-mediated absorption, heat diffusion and thermally induced local IMT successfully explains the resonant pumping on picosecond time scales. Nanoantenna–VO_2_ hybrids with fast, picosecond response and picojoule switching energies enable new directions in all-optical switching, active control of quantum emission^37^ and plasmonic memristor-type devices exploiting nanoscale thermal memory^28, 38^.

## Materials and methods

### Fabrication of VO_2_ thin films

VO_2_ thin films with low surface roughness were fabricated using atmospheric pressure chemical vapor deposition following the methods described elsewhere^35^. In short, VO_2_ films were deposited on boroaluminosilicate glass (Eagle by Corning, St Davids Park, UK) coated with a 30-nm layer of fluorine-doped tin oxide (FTO). The inclusion of this intermediate layer allowed the production of VO_2_ films with low surface roughness and suitable thermochromic transition temperature. FTO films were deposited onto 1-mm boroaluminosilicate glass using a precursor mixture of monobutyltintrichloride (MBTC), oxygen, water and trifluoroacetic acid (TFA). A laboratory-scale atmospheric pressure chemical vapor deposition (CVD) coater was used. The substrate was heated to 600 °C and repeatedly translated underneath a coating head to produce a 30-nm-thick layer. MBTC was heated to 95 °C in a stainless-steel bubbler and introduced to a CVD reactor in a nitrogen carrier gas flow. Water and TFA were mixed to give a 0.1 M solution and introduced to the CVD reactor by flash evaporation in a nitrogen carrier gas flow.

VO_2_ was deposited on top of the FTO layer using a precursor mixture of vanadium chloride and water in a ‘static’ cold wall atmospheric pressure CVD reactor. The substrate was sealed in a reaction chamber, allowing the area to be pressure-purged and backfilled with nitrogen, producing a controlled, oxygen-free environment for deposition. The temperature was increased to the coating temperature, and the reactants were passed parallel over the substrate for 1 min to produce a 50-nm coating. Vanadium chloride was heated to 100 °C in a stainless-steel bubbler and was introduced into the coater in a nitrogen carrier gas flow. Water was heated to 60 °C in a stainless-steel bubbler and was introduced to the coater in a nitrogen carrier gas flow. After film deposition, the sample was cooled to <200 °C before removal from the coater to prevent oxidation of the coating.

### Fabrication of nanoantennas

Gold nanoantennas were fabricated on top of the 50-nm VO_2_ layer using e-beam lithography (JBX-9300FS by JEOL, Tokyo, Japan) at 100 keV with a dose of 650 μC cm^−2^. After development, 45 nm of gold was evaporated followed by lift off in acetone. The width of the antenna arms was fixed at 80 nm, and the length was varied between 160 and 360 nm. A Ti layer of 5-nm thickness was used to improve the adhesion of the gold to the VO_2_. Helium-ion microscopy (HIM) was performed on all arrays under study (Orion, Zeiss, Jena, Germany). Compared with conventional electron microscopy, the helium-ion microscope allows high-resolution imaging of non-conducting samples without charging the substrate.

### Optical experiments

The dielectric function of the VO_2_ film was characterized using variable-angle ellipsometry (Uvisel 2, Horiba, Stanmore, UK)^35^. Antenna experiments were performed using a picosecond pulsed Yb-fiber laser (Fianium, Hamble, UK) with a variable repetition rate between 0.1 and 20 MHz and a fixed pulse duration of 9.2±0.1 ps, as determined using an autocorrelator (FR-103XL, Femtochrome, Berkeley, CA, USA). The oscillator output was amplified using two separate amplifier stages, one of which was used as the pump laser, with a fixed wavelength of 1060 nm, and the second output was used to produce a broadband supercontinuum spanning the range 400–2500 nm. A spectral band with tuneable centre wavelength and 2% spectral width was selected using a double-prism monochromator in subtractive mode. The 1060-nm pump and the continuously tuneable probe were combined onto an 1100-nm long-pass dichroic beam splitter and were focused onto the array using a 0.5 numerical aperture (NA), reflective Cassegrain objective (Edmund Optics, York, UK). All reported energies were measured before the objective, which has a transmission of 82%. Approximately 65% of the transmitted intensity was focused to a waist of 1.4±0.1 μm, while 35% of the intensity was contained in much weaker satellite structures of the Cassegrain point-spread function extending up to several micrometers from the focus. Fluence values were calculated for the central 6±1 μm^2^ focus area taking into account these factors. The absolute transmission measurements and pump–probe spectroscopy were obtained using a combination of optical choppers and lock-in detection. A motorized variable optical density (OD) filter (OD 2.0) was used to scan the pump power between 1 and 100% of the available range, with 100% corresponding to 13 nJ of pulse energy. For experiments without optical pumping, extinction measurements over a larger spectral window from the visible were collected by removing the dichroic splitter. The sample was mounted on a heater stage with a temperature controller capable of maintaining stable temperatures in the range 25–80 °C.

### Numerical modeling

The optical response of the antennas on the VO_2_–glass substrate stack was modeled using FDTD simulations (Lumerical, Vancouver, BC, Canada). Following detailed HIM images (see Supplementary Fig. S3), the antennas were modeled as semi-cylinders with rounded end caps. Three-dimensional near-field absorption profiles were extracted and used as the source distribution for a heat-transport simulation performed using the Multiphysics finite element software (COMSOL 5.1, Cambridge, UK). The resulting temperature profiles were used to generate a three-dimensional contour map of the phase-change response around the antenna, which was subsequently used to calculate the modified antenna resonances. A more extensive description of the theoretical model is given in the Supplementary Section II.

## Results and discussion

### Thermally induced phase transition of antenna–VO_2_ hybrids

To investigate the resonant antenna-assisted pumping of the VO_2_ IMT, arrays of crossed gold nanoantennas were fabricated on top of a VO_2_ film, with antenna lengths varying from 160 to 360 nm in steps of 50 nm (HIM images shown in Supplementary Fig. S2). A continuous VO_2_ film of 50-nm thickness with a roughness of 5 nm, estimated using atomic force microscopy, was used as the substrate for depositing gold nanoantennas using e-beam lithography.

**Figure 1:**
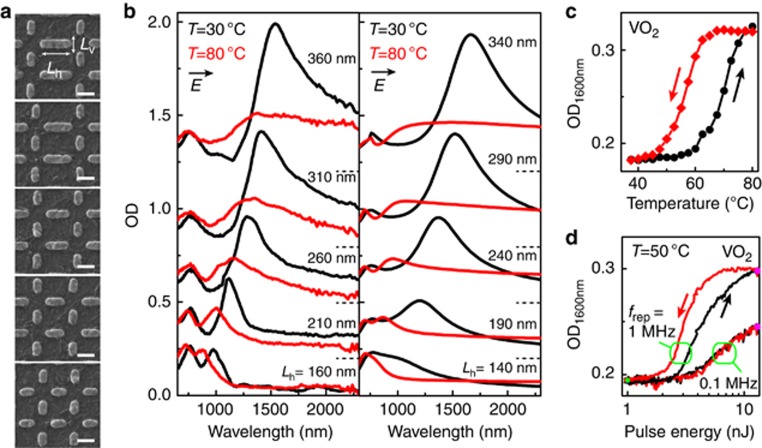
Temperature control of the resonances of the antenna–VO_2_ hybrids. (**a**) Helium-ion microscopy images of antenna arrays corresponding to fixed *L*_v_=160 nm and, from bottom to top, *L*_h_=160, 210, 260, 310 and 360 nm. Scale bars, 200 nm. (**b**) Experimental (left) and calculated (right) extinction spectra (OD) of antenna arrays corresponding to the images of **a**, for horizontal polarization. The response of the bare VO_2_ film and substrate without antennas was subtracted (see Supplementary Information). The sample temperature was set to 30 °C (black lines) and 80 °C (red lines). (**c**) Temperature hysteresis curve for the OD of bare VO_2_ film without antennas, taken at *λ*_probe_=1600 nm. (**d**) Optical pumping hysteresis curve for bare VO_2_, for increasing (black) and decreasing (red) pump energies, taken at repetition rates of 1.0 and 0.1 MHz and a pump wavelength 1060 nm.

Figure 1a shows five selected arrays, where the vertical antenna length *L*_v_ was kept fixed at 160 nm while the length *L*_h_ of the horizontal antennas was varied. This subset represents the behavior of all arrays of antennas, which follow very similar trends (see Supplementary Fig. S5). Figure 1b presents horizontally polarized extinction spectra (OD) for temperatures below (black) and above (red) the critical phase transition. A background spectrum was subtracted from all spectra corresponding to the bare VO_2_ substrate (see Supplementary Fig. S1). At temperatures far below the phase transition, the spectra show pronounced optical resonances in the near-infrared corresponding to the longitudinal half-wave (*λ*/2) antenna mode with a resonance wavelength shifting from 950 to 1500 nm with increasing antenna length. Above the phase transition (red lines, 80 °C) these resonant modes are strongly suppressed. This suppression can be qualitatively understood by the large changes in the optical permittivity of the VO_2_ film^35^, which is characterized by a strong reduction of the real part and an increase of the imaginary part of the permittivity in the infrared. The VO_2_ film acts as a load on the antenna response, resulting in both a blueshift and strong damping of its resonance.

The spectral response at temperatures below and above the phase transition is reproduced well by numerical calculations of the antenna–VO_2_ hybrid, as shown in Figure 1b, obtained using the measured dielectric function of the VO_2_ film, as detailed in the Supplementary Section II. Good agreement is obtained for the shape and amplitude of the spectra both below and above the phase transition, showing that a suppression of the antenna resonance OD by up to 60% is achieved through temperature control of the VO_2_ substrate. A small adjustment of all antenna lengths to 20-nm lower values was made to improve the agreement of the spectral resonance positions at low temperature. Furthermore, for shorter antennas with resonances near the visible range, the calculated antenna resonances are somewhat broadened compared with the experimental data, indicating an overestimate of the local absorption in the VO_2_ substrate in this spectral range. We attribute these small deviations to variation in the optical properties over the film thickness that is not captured fully by the ellipsometry^35^, which provides only the average film properties.

### Optically induced phase transition of antenna–VO_2_ hybrids

The reduction of the antenna OD above the phase transition is opposite to the effect of the VO_2_ film itself, where the OD is increased above the phase transition due to increased absorption in the switched metallic state, as shown in Supplementary Fig. S1. The spectral response shows an isosbestic point (that is, wavelength of constant OD between the two states) at ~1000 nm, with an increasing IMT-induced OD at longer wavelengths. In the following, we choose a probe wavelength of 1600 nm, sufficiently far from the isosbestic point, as representative for the dynamics of the VO_2_ film without antennas. The temperature-dependent OD of the VO_2_ film without antennas is presented in Figure 1c. The VO_2_ film shows a typical hysteresis loop when the temperature is cycled between 25 and 80 °C, as indicated by the arrows. This hysteresis shows that thermal memory is present in the system, that is, the sample response depends on its thermal history.

Next to controlling the sample temperature, the phase transition could be triggered through optical pumping. For this purpose, we fixed the sample temperature to 50 °C, sufficiently below the hysteresis loop of the VO_2_ to allow full relaxation of the sample to its low-temperature, unswitched state. The optical excitation makes use of a picosecond pulsed laser, as described in the Materials and Methods section, producing a train of pulses with variable repetition rate. To study the switching caused by individual pulses over a single cycle, as opposed to accumulated heating effects, it is important that the time between subsequent pump pulses is sufficiently long to allow the sample to recover its unperturbed state without a memory of previous events. Figure 1d shows the response of the VO_2_ film at 1600 nm under excitation by a train of picosecond pump pulses at a repetition rate of 1.0 MHz and a pump wavelength of 1060 nm. The OD was sampled at a pump–probe time delay of 100 ps following pulsed laser excitation, corresponding to a fully developed phase transition. With increasing pulse energy, the OD increases to a level close to the thermal switching amplitude of Figure 1c at the highest available pump energy of 13 nJ, indicating that a complete phase transition is achieved using optical pumping under these conditions. However, when ramping down the power, hysteresis behavior is observed. This hysteresis indicates that thermal memory is still present for pulsed excitation at a 1-MHz repetition rate, and part of the response results from heat accumulated in the sample over subsequent excitation cycles. The hysteresis in the optical pumping cycle is completely suppressed when reducing the repetition rate to 0.1 MHz, where it is evident that single-cycle pulsed excitation only achieves approximately half of the modulation amplitude of Figure 1c.

**Figure 2:**
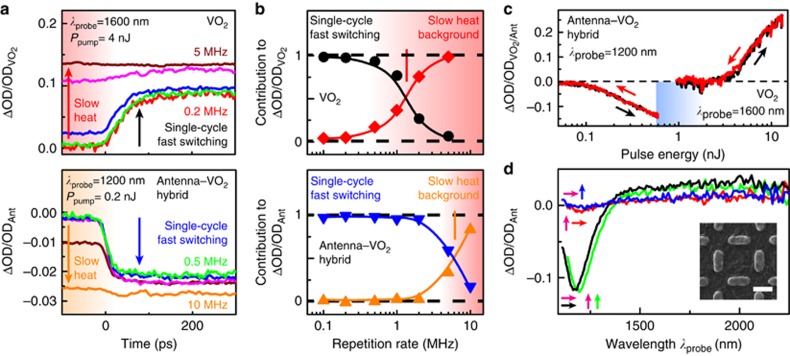
Contributions of the fast picosecond and slow thermal response to the optically driven IMT in VO_2_ and antenna–VO_2_ hybrids. (**a**) Time-dependent response of VO_2_ (top, *λ*_probe_=1600 nm) and antenna array with *L*_v_=*L*_h_=210 nm (bottom, *λ*_probe_=1200 nm) for repetition rates of 0.2 MHz (red), 0.5 MHz (green), 1 MHz (blue), 2 MHz (magenta), 5 MHz (brown) and 10 MHz (orange). The effect before 0 ps corresponds to a slow heat background, with the step at 0 ps corresponding to single-cycle fast switching caused by picosecond phase transition. Pump energy *P*_pump_ for the VO_2_ film, 4 nJ; for the antenna array, 0.2 nJ. (**b**) Relative contributions of single-cycle fast ΔOD_fast_/ΔOD and slow heat background ΔOD_slow_/ΔOD (see text) obtained from the time traces in **a**. The antenna array shows 5× increased operating speed for single-cycle fast switching compared with pure VO_2_. (**c**) Phase-change modulation ΔOD/OD versus optical pulse energy at *λ*_probe_=1200 nm for the antenna array and at *λ*_probe_=1600 nm for the VO_2_ film, both at 0.1 MHz. The blue shaded region indicates the damage threshold for the antenna array. (**d**) Differential nonlinear response spectrum ΔOD/OD_Ant_ for an antenna array with *L*_v_=*L*_h_=210 nm, for different combinations of pump (magenta arrows) and readout (black/red arrows, horizontal, green/blue arrows, vertical) polarizations, at a temperature of 50 °C, 0.1 MHz pump laser repetition rate, and 0.6 nJ pump energy.

The balance between fast, single-cycle switching and slow, accumulated heat effects is investigated in more detail in Figure 2 by studying the dependence on the optical pumping repetition rate. We used a pulse picker to reduce the laser repetition rate in steps from 10 to 0.1 MHz. The top panel of Figure 2a shows the differential transmission ${\text{ΔOD/OD}}_{{\text{VO}}_{2}}$ against the pump–probe delay time for the bare VO_2_ film without antennas. The bottom panel in Figure 2a shows the response measured for the antenna–VO_2_ hybrid array with *L*_v_=*L*_h_=210 nm at the antenna resonance (*λ*_probe_=1200 nm), normalized to the maximum OD of the antenna, OD_Ant_=0.4 (see Supplementary Fig. S4). The pump energy of *P*_pump_=0.2 nJ was limited by the damage threshold at the highest repetition rates. The relevant dynamics did not depend on the pulse energy.

A picosecond, fast component is identified by the step in the ΔOD/OD response at ~0 ps. At high-repetition rates, the amplitude of this fast, picosecond component is reduced, whereas instead the ΔOD/OD response is governed by a flat, stationary response that does not depend on the picosecond dynamics. The balance between these two effects is best illustrated by plotting the relative contributions to the total response shown in Figure 2b, where the contributions are defined as ΔOD_slow_/ΔOD and ΔOD_fast_/ΔOD. Here ΔOD is the total change, and ΔOD_slow_ and ΔOD_fast_ are the slow and fast components, identified by the ΔOD before and after 0 ps, respectively. For low-repetition rates, the response is entirely governed by the fast, picosecond component corresponding to excitation by a single-pump pulse at every cycle. At higher repetition rates above a critical threshold (indicated by the vertical line), the stationary thermal background dominates, and the fast, picosecond component is suppressed to zero. The stationary background corresponds to an increased temperature of the sample around the excitation area, which is built up over many pumping cycles because the sample is not completely relaxed between subsequent pulses. Because both the background and picosecond effects act on the same IMT, an increase of the slow effect results in suppression of the fast component because the sample is maintained in the switched state by the accumulated heat. For the VO_2_ film without antennas, this crossover from a fast picosecond response (black dots) to the slow heat background signal (red diamonds) occurs at an ~1 MHz repetition rate, indicating that the typical recovery time of the system is ~1 μs. For the antenna–VO_2_ hybrid system, the crossover from picosecond to slow heat background response occurs at ~5 MHz, five times higher frequency than for the VO_2_ film. Thus, the typical recovery time of the hybrid system is ~200 ns.

The response of the antenna–VO_2_ hybrid is characterized by a strong reduction in the optical switching energy. Figure 2c compares ΔOD/OD_Ant_ for the *L*_v_=*L*_h_=210 nm antenna–VO_2_ hybrid with ${\text{ΔOD/OD}}_{{\text{VO}}_{2}}$ of the bare VO_2_ film against pulse energy at a repetition rate of 0.1 MHz. While the bare VO_2_ shows an onset of IMT switching for amplitudes >2 nJ, the antenna–VO_2_ hybrids show an onset at ~100 pJ, which is a 20 times lower pulse energy. Irreversible damage to the antenna–VO_2_ hybrids is caused above 800 pJ, limiting the maximum modulation for this configuration to ~15%. The VO_2_ film itself reaches similar modulation contrast values of up to 20% however, this requires a much higher pulse energy of 13 nJ.

### Antenna-assisted resonant pumping of the phase transition

The fivefold reduction in thermal recovery time and >20 times lower switching energy of the antenna–VO_2_ hybrids suggests a new excitation pathway distinct from bulk switching of the VO_2_ film without antennas. To further explore the contribution of antenna-assisted mechanisms in the antenna–VO_2_ hybrid response, we measured the switching response while independently controlling the polarizations of both excitation and readout. This strategy was used successfully in previous studies for antennas on an ITO substrate^36^. Figure 2d shows the spectrally resolved ΔOD normalized to OD_Ant_ measured on the *L*_v__,h_=210 nm antenna–VO_2_ hybrid array for the four different combinations of pump and probe polarizations, as indicated by the pairs of arrows, with the first (magenta) and second (colored) arrow, indicating pump and probe polarizations, respectively. The pumping was performed at a 0.1 MHz repetition rate and 0.6 nJ energy at 1060 nm pump wavelength. A large modulation amplitude is observed only for the condition, where the pump and readout polarizations are parallel to each other. For perpendicular polarizations, no switching response is observed. Given that perpendicularly oriented antennas are positioned within 100 nm from each other in the array, the lack of cross-interaction of the antenna-mediated IMT is remarkable and shows experimentally that the induced effects are limited to only a nanoscale-sized volume around the antenna–VO_2_ hybrids, which can be specifically addressed by the combination of the polarization and wavelength of the excitation laser.

**Figure 3:**
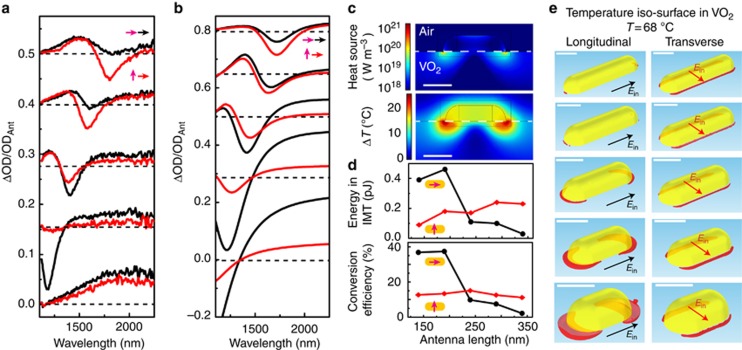
The model shows that the predominant phase-change response is caused by antenna-assisted absorption in the VO_2_ at plasmonic hotspots. Experimental (**a**) and calculated (**b**) switching response ΔOD/OD_Ant_ of the antenna–VO_2_ hybrids for parallel (black lines) and perpendicular (red lines) polarizations for excitation and readout for (bottom to top) antenna arm lengths *L*_h_=*L*_v_=160, 210, 260, 310 and 360 nm (calculated antennas are 20 nm shorter). Dashed lines indicate vertical offsets. (**c**) Calculated heat source distribution (top panel) in antenna–VO_2_ hybrid with 190 nm antenna length during the pump laser pulse, calculated using the full electrodynamic model of local absorption, and the temperature increase Δ*T* calculated by including the time-dependent heat diffusion model for 9.2-ps pump duration. Scale bars, 100 nm. (**d**) Calculated energy contained in the IMT regions and conversion efficiency (% of incident energy) versus antenna length and pump polarization (arrows) for a single antenna. (**e**) Temperature iso-surfaces for the critical phase transition temperature, *T*=68 °C, for antenna lengths from 160 to 360 nm (bottom to top) and longitudinal (left row) and transverse (right row) polarizations. Scale bars, 100 nm.

Experiments performed on different arrays of antenna–VO_2_ hybrids, corresponding to different combinations of lengths *L*_h_ and *L*_v_, show the response when tuning the system from resonant to non-resonant pumping conditions. Figure 3a shows the results for arrays of crossed antennas with equal arm lengths *L*_h_ and *L*_v_ tuned (bottom to top) from 160 to 360 nm. The polarization-dependent response is shown for the pump and readout polarizations that are parallel (black curves) and perpendicular (red curves). The strong IMT response around the plasmon resonance for parallel polarizations, which is most clearly observed for the 210-nm length antennas corresponding to Figure 2d, disappears with increasing arm length. For crossed polarizations, the nonlinear ΔOD response increases as the antenna length is increased.

### Model of antenna-assisted phase transition

These trends are reproduced well by the detailed numerical calculations plotted in Figure 3b. The physical model underlying these calculations is presented in Figure 3c–3e. First, the near-field absorption profile around the antenna was calculated based on full electrodynamic calculations of the near-field intensity. The top panel of Figure 3c shows resulting maps for an *L*=190 nm antenna and incident light polarized along the antenna axis, for an excitation power corresponding to an integrated energy of 300 pJ (fluence of 2.75 mJ cm^−^^2^) over the pulse duration. The calculated local absorption profile was used as a quasi-stationary heat source distribution for the heat-transport equation, resulting in the temperature profile shown in the bottom panel of Figure 3c. To separate the different contributions to the total temperature rise originating from energy absorbed in the gold antenna and in the VO_2_ film, we independently evaluated the role of the antenna as a heat source (results shown in the Supplementary Fig. S8), and found that absorption outside the plasmonic antenna dominates the heat source distribution under conditions of resonant pumping and nearly completely explains the temperature increase without requiring additional energy transport from inside the nanoparticle into the VO_2_ layer.

From the calculated local temperature profile, nanoscale volumes can be extracted, in which the temperature is sufficiently high to induce the VO_2_ IMT. Figure 3e shows the resulting contours, plotted in red, for antennas with lengths increasing from 140 to 340 nm (the length of the model antennas corresponds to the experimental cases from 160 to 360 nm). Both the longitudinal and transverse pump polarizations are shown for each antenna. For longitudinal pump polarization, resonant pumping results in pronounced pockets of IMT response around the end caps of the antenna for the shortest antennas. For longer antenna lengths, the effect of resonance is weaker, and the critical temperature threshold is reached within a much smaller volume. In comparison, the transverse pump polarization shows approximately the same effect for all antenna lengths. This contribution is caused by off-resonance absorption of the transverse plasmon mode, which causes a narrow band of IMT that increases linearly in length as the antenna length is increased.

The optical spectra in Figure 3b were obtained by including the calculated IMT volumes in the electrodynamic simulation, using the known dielectric response of the switched VO_2_. Our microscopic model satisfactorily explains the two trends for the longitudinal and transverse excitation conditions, corresponding to the resonant pumping of the IMT for longitudinal polarization and the non-resonant increase for transverse polarization. The model predicts an effective switching energy of up to 0.46 pJ per antenna at resonance, as shown in Figure 3d. Up to 39% of the total absorbed energy is converted into the VO_2_ phase transition, making the resonant, antenna-assisted absorption mechanism a potentially highly efficient process for device applications. For the pumping of the transverse resonance, the model calculates energy conversion efficiencies in the range of 12–15%.

**Figure 4:**
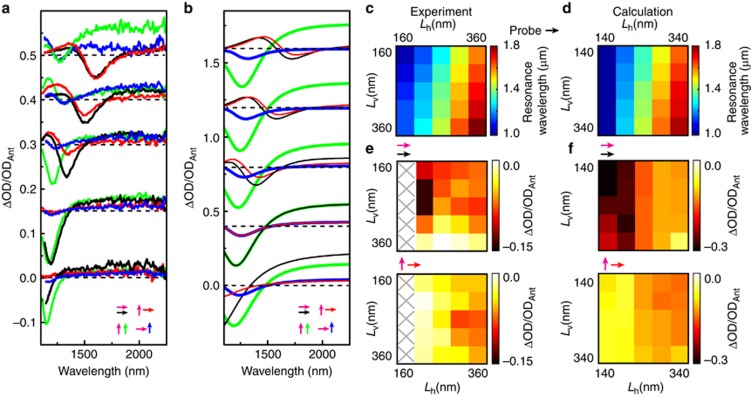
Full investigation of the multifrequency antenna–VO_2_ hybrids shows resonant contribution in antenna-mediated IMT response. (**a**) Experimental and (**b**) calculated switching response ΔOD/OD_Ant_ of antenna–VO_2_ hybrids for fixed *L*_v_=210 nm and, bottom to top, varying *L*_h_=160, 210, 260, 310 and 360 nm (calculated antennas are 20 nm shorter). Arrows indicate pump (magenta) and probe (black/red arrows, horizontal, green/blue arrows, vertical) polarizations. (**c** and **d**) Experimental (**c**) and calculated (**d**) resonance positions of the horizontal (top panel) antennas in multifrequency antenna arrays with combinations of *L*_h_ and *L*_v_ (see Supplementary Fig. S2 for helium-ion microscopy images). (**e** and **f**) Experimental (**e**) and calculated (**f**) normalized amplitude ΔOD/OD_Ant_ for the horizontal readout (probe, black/red arrows) and for two pump polarizations, as indicated by magenta arrows. Crosses indicate arrays for which the resonance lies outside the spectral window of the probe.

The results in Figure 3 show that the switching spectra for cross-antenna arrays with equal lengths can be explained using the antenna-assisted IMT response of single antennas without cross-coupling. The case of unequal antenna lengths is investigated in Figure 4. Figure 4a shows the full polarization response for four combinations of pump and probe polarizations for antennas with fixed *L*_v_=210 nm and *L*_h_ varying from 160 to 360 nm. Each ΔOD spectrum was normalized to the OD_Ant_ of the corresponding antenna resonance. The corresponding calculated response is shown in Figure 4b. Both experiment and theory show good agreement in the spectral positions and line shapes of the ΔOD switching response, resembling that of the uncoupled single rods. The only deviation is for the resonant excitation of the fixed vertical antenna (green curves), which shows a suppression and change in shape for the longest horizontal antenna lengths.

The global trends over the 25 (5 × 5) different arrays are illustrated by the color maps shown in Figure 4c–4f. Figure 4c and 4d shows the spectral resonance positions extracted from the linear extinction spectra (see Supplementary Figs. S4 and S6). The maximum negative ΔOD/OD_Ant_ amplitudes are shown in Figure 4e and 4f for two of the polarization combinations. The other two polarization cases can be obtained by mirroring the matrices along the diagonal. For the shortest 160-nm length antennas, the resonance lies below the accessible spectral range; therefore, we discarded these data points (crossed out).

The diagonal components of the matrices correspond to the equal length antenna arrays in Figure 3a and 3b, and show good agreement between experiment and theory. For the off-diagonal components, the transverse (cross-polarized) pumping conditions are reproduced for all combinations and show little dependence on the ratio of the horizontal and vertical antenna lengths (vertical bands of equal modulation intensity). For the longitudinal excitation (parallel polarizations), much stronger co-dependence on the antenna parameters is observed for the experimental case than for the calculations, as was observed in Figure 4a. The strongest overall modulation response is found for the *L*_v_=*L*_h_=210 nm array, and the presence of longer antennas in the cross-direction reduces the modulation depth in the experimental data but does not affect the calculated response. Thus, the experimental data indicate stronger cross-interaction effects between antennas, which are not yet fully captured by our model. These variations correspond to subtle differences in the microscopic energy distribution, which may be fine-tuned in future work. Overall, we find that the simple localized absorption picture provides a good qualitative and semi-quantitative description of the observed behavior.

### Thermal latching optical memory of antenna–VO_2_ hybrids

**Figure 5:**
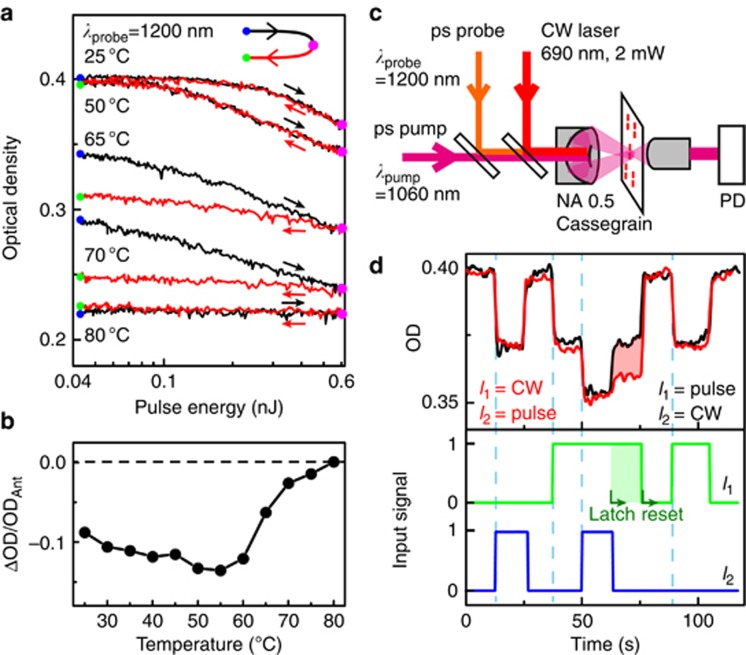
Thermal latching and memory functionality of antenna–VO_2_ hybrids. (**a**) Optical density of the antenna–VO_2_ hybrid with *L*_v_=*L*_h_=210 nm while scanning the pulse energy up (black line) and down (red line) for background temperatures from 50 to 80 °C. Data were taken at 1200 nm and for parallel pump and readout polarizations at 0.1 MHz. (**b**) Differential response ΔOD/OD_Ant_ versus background temperature for the same experimental conditions as in **a**, for *P*_pump_=0.6 nJ. (**c**) Experimental setup for the combined pulsed and CW pumping experiment. (**d**) Combined effect of two input pump sequences corresponding to 1 ps and one CW input. Sequence shows individual effects of both input *I*_1_ and *I*_2_ (bottom panel) and the combined effect of the two signals, including a memory latch (indicated by shaded area). Memory functionality is only observed for the CW control input *I*_1_, whereas picosecond pulses do not provide latching functionality.

The above investigation shows that it is possible to achieve local IMT switching through resonant optical pumping of VO_2_ using nanoscale hotspots. The local pumping significantly reduces the required optical power, thermal load and recovery time of the device. These characteristics are of interest for applications in optical memory, memristors, switches and modulators. The memory functionality of antenna–VO_2_ hybrids is based on the thermal hysteresis characteristics, which we minimized by using pulsed laser excitation at sub-MHz repetition rates. However, memory functionality can be introduced by raising the base temperature into the hysteresis regime^28^. Figure 5a shows the dependence of the optically induced state on the base sample temperature, as set by the external heater stage. At room temperature, picosecond optical switching is fully reversible and can be cycled over millions of times per second. At elevated temperatures above 55 °C, the system is latched to a value determined by the downward branch of the hysteresis loop of Figure 1c. This latching effect for the case of resonant plasmonic pumping is nontrivial because it involves maintaining the integrity of the small, nanoscale pockets of switched VO_2_ around the antenna end caps. In our experiments, we did not observe degradation of the latched state over typical time scales of minutes. At sample temperatures above the IMT (80 °C in Figure 5a), optical pumping does not induce further effects because the whole sample is already switched thermally by the heater stage.

In the differential ΔOD/OD_Ant_ response (Figure 5b), taken at the pulse energy of 0.6 nJ, the thermal latching effect is observed as a reduction of the picosecond transient signal to zero as the system crosses the hysteresis regime of the IMT. Furthermore, while the largest signal amplitudes are obtained just below the hysteresis regime at ~50 °C, a modulation response is obtained all the way down to room temperature, showing that operation of antenna–VO_2_ hybrids for picosecond switching is possible without requiring an elevated base temperature.

In addition to setting the base temperature, latching of the phase transition could also be achieved through continuous-wave optical pumping of the sample. Figure 5c shows the experimental arrangement, where a continuous wave (CW) diode laser at a wavelength of 690 nm was used to achieve continuous thermal excitation at a ΔOD level comparable to that of the picosecond laser-induced response. We tested the latching effect using two pulse sequences, where the CW and picosecond pulsed pump lasers were combined to provide an additive effect on the IMT response of the readout beam. In the first scheme, intensity sequence *I*_1_ was applied to the CW laser and sequence *I*_2_ was applied to the picosecond laser. In this sequence, the switch was prepared in the trigger state at 40 s using the CW laser (*I*_1_), after which the memory was triggered by the picosecond laser (*I*_2_) at 50 s. When the picosecond signal *I*_2_ was switched off at 65 s, the system remained in the latched state until the removal of the CW control signal at 80 s. Similar to thermal latching using the heater stage, the optically latched state was very stable over minutes, and the fluctuations in Figure 5d mainly reflect stability of the microscope setup itself. When *I*_1_ and *I*_2_ are reversed, that is, *I*_1_ corresponds to the single-shot reversible excitation, this latching effect is no longer obtained because the base temperature is not retained for pulsed excitation. Thus, picosecond pulsed excitation and CW thermal excitation with the same optical response provide different memory functionalities and can be used in combination to achieve different switching operations.

### Discussion

It is of interest to compare our results obtained in the picosecond regime with other works using continuous-wave or ultrafast (sub-100 fs) pulsed excitation. The critical pulse energies for the antenna–VO_2_ hybrid and the bulk VO_2_ translate to fluences of ~0.9 mJ cm^−^^2^ and 18 mJ cm^−^^2^, respectively. Continuous-wave experiments show a threshold for the VO_2_ IMT of ~100 mW cm^−^^2^ (Refs. 28 and 33). However, this mechanism involves slow heating of both the VO_2_ and substrate to the critical temperature over hundreds of milliseconds^33^. For sub-100 fs excitation, the critical fluence amounts to 4.7 mJ cm^−^^2^ for bulk VO_2_^18, 39, 40^. The fluences in our work are therefore higher than the values reported for nonthermal excitation of VO_2_ domains, which benefit from highly nonequilibrium effects to cross the energy barrier for the IMT. While the use of picosecond pulses does not benefit from the nonequilibrium physics of ultrafast lasers, there is a strong motivation for device applications to exploit efficient picosecond mechanisms for optical devices, such as those based on the fast thermal response of plasmonic nanoantennas^34^. Because the pump area contains ~60 of the resonant antennas, the experimental switching energies between 100 and 600 pJ correspond to 1.6 and 10.0 pJ per antenna, closely matching our numerical model calculations. Thus, picojoule single-antenna switches are feasible using this approach.

In comparison, the reported switching fluence of chalcogenide phase-change materials using femtosecond laser pulses is 140 mJ cm^−2^ (Ref. 13), corresponding to 1.5 nJ pulse energy for a full set-reset cycle. In particular, the reset pulse in non-volatile GST phase-change memory locally exposes the device to temperatures above the melting point of 620 °C, resulting in a significant thermal load and crosstalk, which can pose limitations in practical applications. In contrast, the temperature in picosecond antenna–VO_2_ hybrids never rises far above the phase transition of 68 °C and is therefore friendlier toward integration into nanophotonic devices. Given that a single picojoule pulse induces persistent IMT switching for several hundreds of nanoseconds, the average power requirement to sustain a single-antenna–VO_2_ hybrid memory can be estimated as ~10–100 μW. Thus, antenna–VO_2_ hybrids are expected to be competitive with GST phase change, especially for short-term memory applications with cycling times shorter than 1 ms.

In addition to the thermally mediated phase transition, recent studies have proposed contributions of ultrafast plasmon-induced hot electrons^31, 32^. These additional effects are more likely to play a role for much shorter laser pulses in the 100 fs range, that is, much shorter than the 9.2 ps pump laser used in this work. Our modeling based on the measured optical constants predicts absorption primarily in the VO_2_ itself and reproduces our experimental results without requiring the additional mechanism of hot-electron excitation and escape through the gold–VO_2_ interface. Therefore, we have not considered these effects in our interpretation.

## Conclusion

We have demonstrated that resonant pumping of plasmonic nanoantenna–VO_2_ hybrids results in high-repetition, fully reversible switching of the insulator-to-metal phase transition. The use of plasmonic near-field enhancement and hybrid antenna–VO_2_ response results in an order of magnitude lower switching energy and faster cycling time than bulk VO_2_ without antennas. Moreover, the antenna-assisted process allows precise, local control of the VO_2_ optical properties down to the nanoscale. These properties render antenna–VO_2_ hybrids of great interest for application in optical modulation and fast picosecond control of nanoscale devices. As a result of the large difference in switching energy between antenna-mediated and bulk VO_2_, the antenna–VO_2_ hybrid response can be selectively excited without inducing a background response from the VO_2_ film itself. Optical experiments show that fully reversible switching of antenna resonances with over two million cycles per second is possible using resonant pumping schemes. The insulator-to-metal phase transition mediated by local pumping of a plasmon resonance does not influence the resonance of a perpendicular nanoantenna positioned <100 nm from the modulated antenna. Further improvement of the response may include optimization of the design, including selective deposition of VO_2_ in the direct surroundings of the antenna, embedding of antennas inside a VO_2_ matrix and improved cooling using substrates and coatings with high-thermal conductivity.
