# Supplementary material for: Antenna-assisted picosecond control of nanoscale phase transition in vanadium dioxide
Source: Light Sci Appl. 2016 Oct 21;5(10):e16173–. doi: 10.1038/lsa.2016.173 (PMC6059831; doi:10.1038/lsa.2016.173)
Supplement: Supplementary Information [file lsa2016173x1.pdf]

Supplementary Information: Antenna-assisted picosecond control of nanoscale phase-transition in vanadium dioxide.

Otto L. Muskens,[1] Luca Bergamini,[2,3] Yudong Wang,[1,4] Jeffrey M. Gaskell,[5] Nerea Zabala,[2,3] C. H. de Groot,[4] David W. Sheel,[5] and Javier Aizpurua[3]

[1)] *Physics and Astronomy, Faculty of Physical Sciences and Engineering, University of Southampton, Highfield, Southampton SO17 1BJ, UK*[a)]

[2)] *Department of Electricity and Electronics, FCT-ZTF, UPV-EHU, Bilbao 48080, Spain*

[3)] *Materials Physics Center, CSIC-UPV/EHU and DIPC, San Sebastian 20018, Spain*

[4)] *Nano Group, Faculty of Physical Sciences and Engineering, University of Southampton, Highfield, Southampton SO17 1BJ, UK*

[5)] *Materials and Physics Research Centre, University of Salford, Manchester, M5 4WT, UK*

I. EXPERIMENTAL CHARACTERIZATION AND SPECTROSCOPY

A. Optical density of VO_2 substrate without antennas

Figure S1a shows the optical density (OD) of the 50 nm VO_2 film used in the experiments, for temperatures below and above the phase transition at 68 °C. The sample shows an isosbestic point (wavelength at which the absorbance does not change) around 1000 nm wavelength. The experimental spectra furthermore show a number of weak (OD<0.05) thin-film Fabry-Perot fringes. Overall, the level of absorption and the isosbestic point around 1000 nm wavelength are reproduced by the numerical model as shown in Figure S1b, using experimental values of the dielectric function of VO_2 taken from spectroscopic ellipsometry. Figure S1c and d shows the response of the VO_2 film associated with the picosecond insulator to metal transition (IMT). The spectral response following switching at a pump energy of 13 nJ is shown in Figure S1c for a delay time of 100 ps. Clearly, the signal increases strongly from the isosbestic point toward the infrared, which has motivated our choice for using 1600 nm as the readout wavelength for the VO_2 phase transition in the main text (the signal to noise ratio of the combined supercontinuum laser and detection electronics deteriorates further toward the IR). The time dynamics of the phase transition shows the same behavior for wavelengths of 1200 nm and 1600 nm as illustrated in Figure S1d.

B. Helium-ion microscopy images of nanoantennas fabricated on top of the VO_2 thin-film

Figure S2 shows an overview of all the arrays used in our experimental studies. The lengths of the horizontal and vertical antennas, L_h and L_v respectively, was varied to obtain multifrequency crossed antenna arrays where the resonance wavelengths could be independently tuned. Overall, the underlying VO_2 is of sufficient smoothness to allow high-quality e-beam nanofabrication using standard lift-off techniques. Figure S3 shows a detailed helium-ion microscopy image of a number of antennas, revealing clearly the polycrystalline nature of both substrate and antennas, with grain sizes in the range of several tens of nm. The antennas have a semi-cylindrical shape with rounded end-caps, an show a an ambiguity in exact dimensions of ±10 nm typical for the e-beam processing and the polycrystalline morphology of the gold.

C. Optical spectra and pump-probe response of all antenna-VO_2 hybrid arrays

A complete overview of all the spectroscopic experiments on the multifrequency arrays is shown in Figures S4 and S5, following the layout of the helium-ion microscopy images, i.e. shortest antenna lengths top left, to longest antenna lengths bottom right. The antenna spectra in Figure S4 show a pronounced evolution of optical resonances from 950 nm for the shortest, 160 nm lengths, to 1800 nm for the longest, 360 nm antennas. The resonance at 750 nm

[a)] Electronic mail: O.Muskens@soton.ac.uk

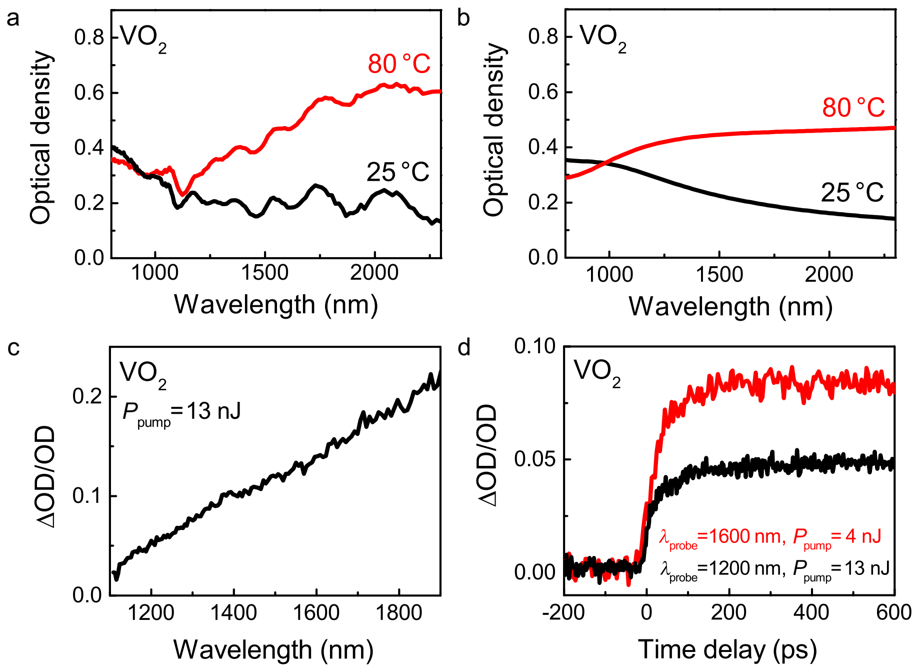

FIG. S1: Experimental (a) and calculated (b) optical density of 50 nm thick VO_2 film without antennas, for temperatures of 25 °C and 80 °C set using a heater stage. (c) Experimental pump-probe response ΔOD/OD for VO_2 film, for pulsed excitation at 1060 nm pump wavelength and pulse energy of 13 nJ, repetition rate 0.1 MHz. The spectrum was collected for a pump-probe time delay of 100 ps. (d) Time-dependent modulation ΔOD/OD for probe wavelength of $\lambda_{\mathrm{probe}} = 1200$ nm, pulse energy $P_{\mathrm{pump}} = 13$ nJ (black curve), and for $\lambda_{\mathrm{probe}} = 1600$ nm, pulse energy $P_{\mathrm{pump}} = 4$ nJ (red curve).

corresponds to the transverse plasmon resonance and does not depend much on the precise configuration of the array. Additional to the length tuning, a redshift of the longitudinal modes is observed due to coupling effects within the array, as the orthogonal antenna is increased in length. This trend is most clearly observed in the extracted peak positions shown in Figure 4a of the main text.

The nonlinear differential signals ΔOD are presented in Figure S5 again for the corresponding arrays of Figure S2. Here all four combinations of pump and probe polarizations were investigated, in order to explore the combined effects of different excitation and readout configurations and multifrequency arrays. In previous work this strategy was used successfully to extract effects of cross-interactions between antennas mediated by a nonlinear substrate[1]. In the antenna-VO_2 hybrid response, such cross-interactions between closely spaced antennas is virtually absent, demonstrating the highly localized nature of the phase-change response around the antennas. The results from Figure S5 were used to extract dip-to-peak values in the modulation associated with the antenna resonances, which were subsequently normalized to the maximum of the optical density spectra of Figure S4 to obtain the relative modulation amplitude $\Delta\mathrm{OD}/\mathrm{OD}_{\mathrm{max}}$. The results for this analysis are presented in Figure 4b of the main text.

II. SIMULATION DETAILS

To obtain the theoretical results shown in Figures 1, 3, 4 of the main text and in Figures S1b, S6, S7 of this Supplementary Information material, we exploited a combination of FDTD and FEM simulations. The OD and ΔOD spectra of the arrays (Figures 1b, 3b, 4c-4d, S1b, S6 and S7) were calculated using the FDTD method, whereas the temperature profiles and the consequent shape of regions around the nanoantennas where the VO_2 changes its phase under optical pumping were obtained from FEM simulations (Figure 3c-3e).

A. OD and ΔOD spectra: Finite-Difference Time-Domain (FDTD)

According to the experimental setup we modelled the system with the bare VO_2 film as a 50 nm-thick VO_2 layer on top of a glass substrate and embedded in air. The antenna-VO_2 hybrid is simulated by placing gold nanoantennas on

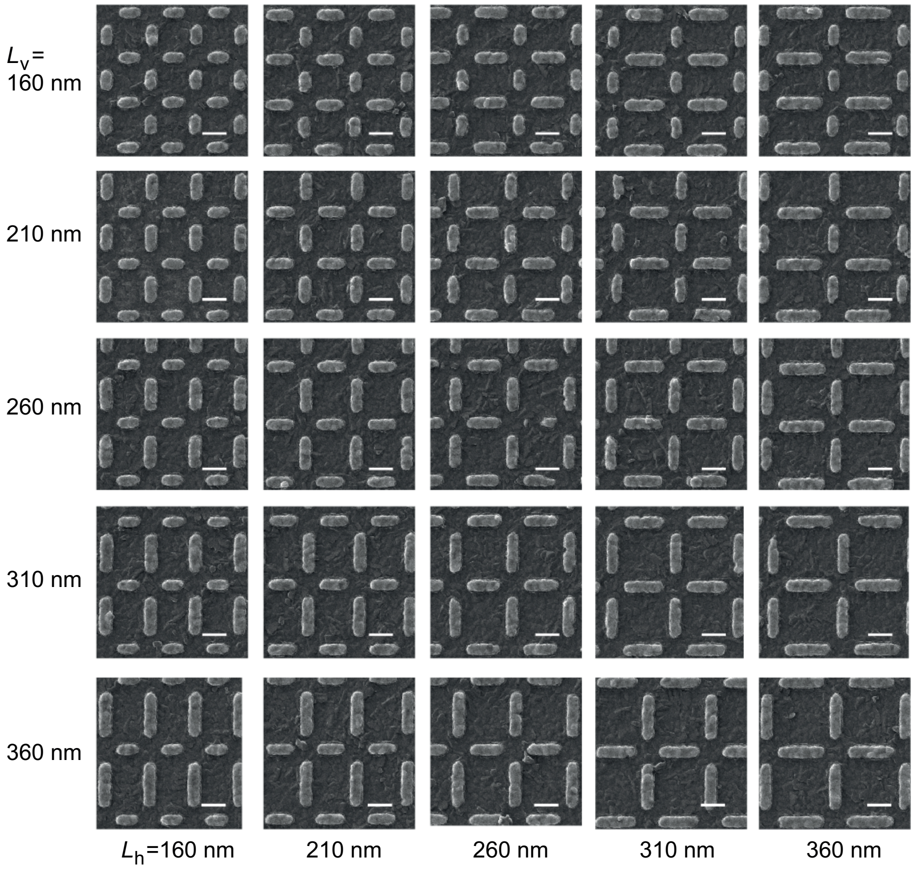

FIG. S2: Helium-ion microscopy images of gold crossed antenna arrays on a 50 nm thin VO_2 film, for antenna lengths ranging from 160 nm to 360 nm in steps of 50 nm. Both the horizontal and vertical antenna lengths are varied independently, resulting in a 5 × 5 parameter space of arrays. Scale bars, 200 nm.

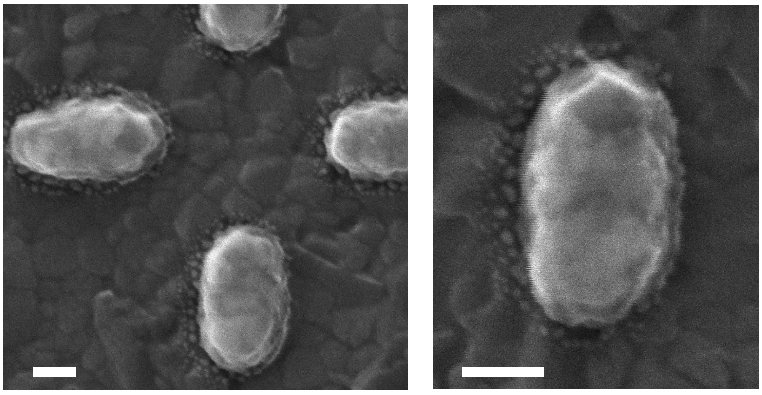

FIG. S3: Helium-ion microscopy images of details of the antenna arrays with $L_v = L_h = 160$ nm. Scale bars, 50 nm. The antennas show a rounded shape approximately described by a semi-cylinder with rounded end caps.

top of the VO_2 film in a cross-arrangement reproducing the experimental design. Each nanoparticle is characterized by a width (short-axis) of 86 nm, a height of 50 nm and a length (L, long-axis) equal to 140, 190, 240, 290 or 340 nm, depending on the sample (values which correspond in the label $x_i y_j$ to the number $i, j = 1, 2, 3, 4, 5$, respectively). All antenna lengths were taken 20 nm smaller than the experimental ones, in order to improve agreement in the optical resonance positions. This small difference lies within the experimental accuracy of the e-beam nanofabrication. The half-rod shape, as well as the size, has been chosen to best match the topological (see Figures S2 and S3) and optical features of the experimental antennas.

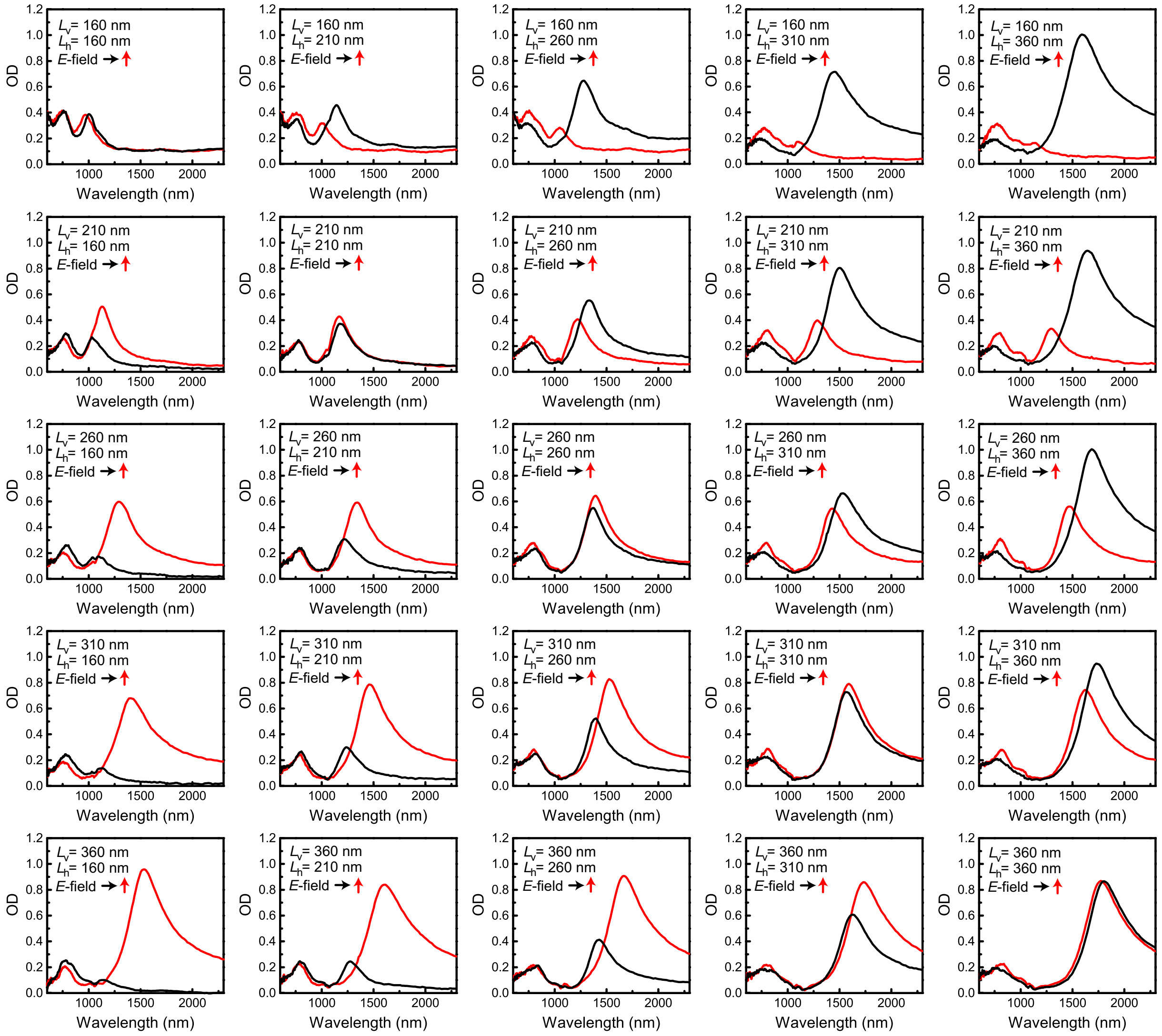

FIG. S4: Extinction spectra (OD) of antenna arrays corresponding to the parameters of Figure S2, for horizontal (black curve) and vertical (red curve) polarizations.

From the optical viewpoint the refractive index of the glass substrate was taken equal to 1.45, while the dielectric function of the nanoantennas was obtained from an interpolation of the experimental data of gold by Johnson and Christy[2]. The dielectric function of the VO_2 film below and above the phase transition temperature ($\approx$ 68 °C) was achieved by using variable-angle spectroscopic ellipsometry taken on the same sample as used in the antenna experiments.

The incident radiation was modelled as a plane-wave impinging perpendicularly onto the sample surface, with an electric field aligned either to the long or short axis of the antennas. To calculate the OD and ΔOD spectra we exploited the FDTD method implemented in the commercial software Lumerical Solutions[3]. The transmission spectrum (T), obtained by means of a "Frequency-domain field and power" monitor placed beneath the substrate, was then elaborated to yield the OD through the relation OD$= -log_{10}T$. The OD spectra shown in the Figures were obtained by subtracting the OD of the VO_2 (black curve in Figure S1b), to mark off better the optical behavior of the antennas. The theoretical black curves of Figure 1b and the curves of Figure S6 are achieved with this setup by employing the VO_2 complex refractive index of its insulator phase, while the red curves in Figure 1b were obtained by using the complex dielectric function of the metal phase of the VO_2 film. As to the ΔOD spectra (Figures 3b, 4c-4d, S7), they were calculated as the difference between the OD of the array before and after the pumping: the former is calculated on the basis of the usual model of the antenna-VO_2 hybrid with the insulating VO_2 film (as above-mentioned), whereas the latter is achieved by switching the VO_2 permittivity to the metal phase in specific regions of the film around the nanoantennas (red hot-spots in Figure 3e of the main text). These hot-spots are

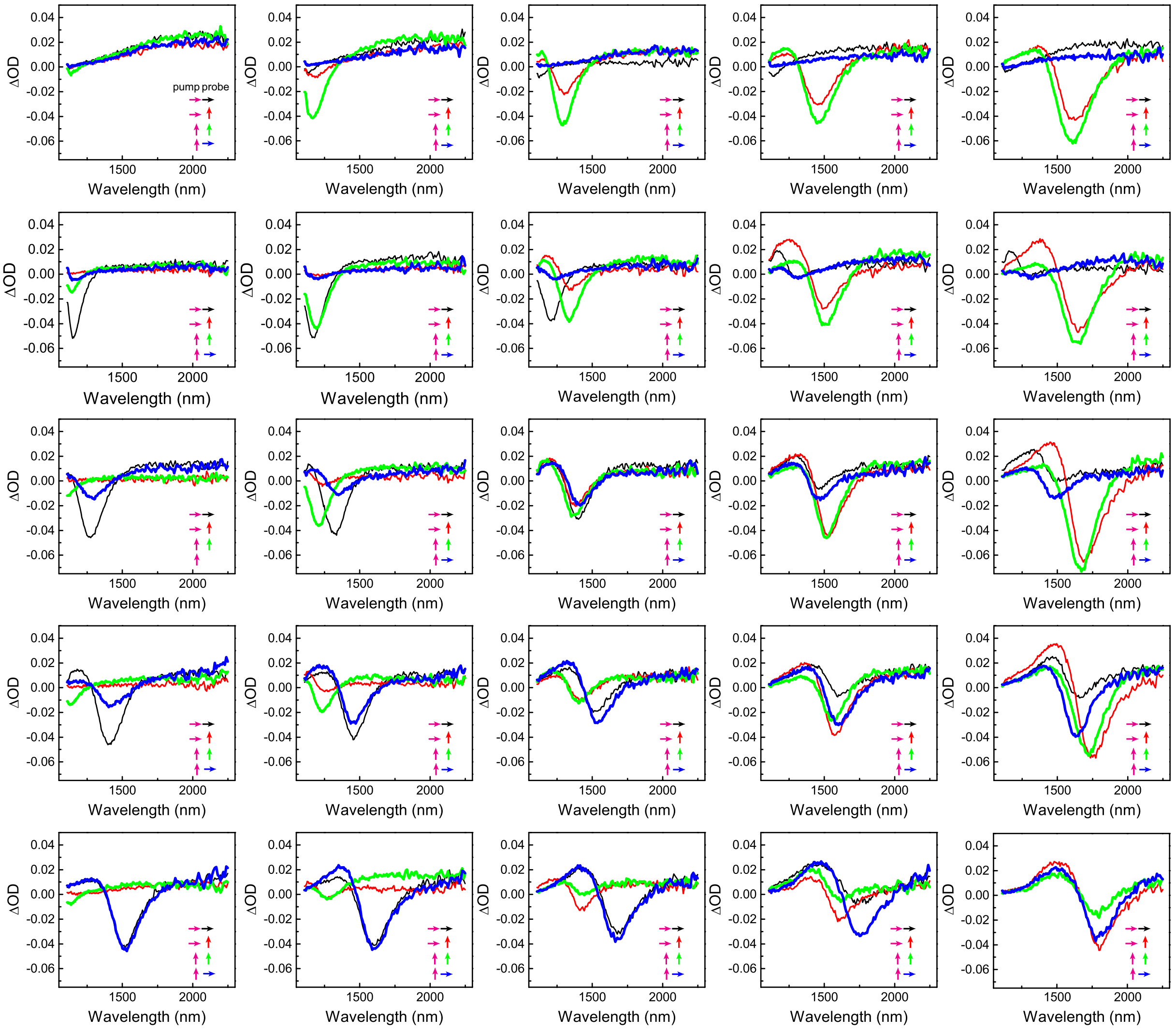

FIG. S5: Differential ΔOD spectra of antenna arrays corresponding to the parameters of Figure S2.

identified according to FEM simulations (see discussion later on) as an effect of the pumping.

B. Pump-induced IMT hot-spots in the VO_2 film: Finite Element Method (FEM)

FEM simulations have been performed via the COMSOL Mutiphysics software[4] to infer the regions inside the VO_2 film where the pump-induced transition in optical properties occurs. This effect is determined by the temperature distribution in the system at the probing time, which involves the solution of the diffusion equations for the heat produced by the metal NPs and the absorbing film. For the sake of simplicity, here we simulated an isolated antenna on top of a VO_2 dielectric film lying on a glass substrate: this holds provided that the antennas are far enough from each other in the array configuration, as it is ensured by the interparticle face-to-face distance of 180 nm. For each antenna size we performed two simulations, one with the incident electric field oscillating along the long-axis (longitudinal polarization) and one along the short-axis (transverse polarization) to embrace both the horizontal and vertical pumping case of all the samples. For instance, by simulating the longitudinal-polarized and the transverse-polarized pumping of the 190 nm long antenna we can deduce the effect around the horizontal and the vertical antennas, respectively, when a pumping electric field is horizontally applied see Figure 3c in the main text.
The FEM simulations consist of two-steps. First, an one-frequency optical simulation is carried out to obtain the total electric field in the system, which is necessary to know the heat source distribution. This is realized through the use of the “Electromagnetic Waves, Frequency Domain” COMSOL module and a “Frequency Domain Study”. The wavelength and intensity of the incoming plane-wave, impinging perpendicularly on the film-substrate stack, were set

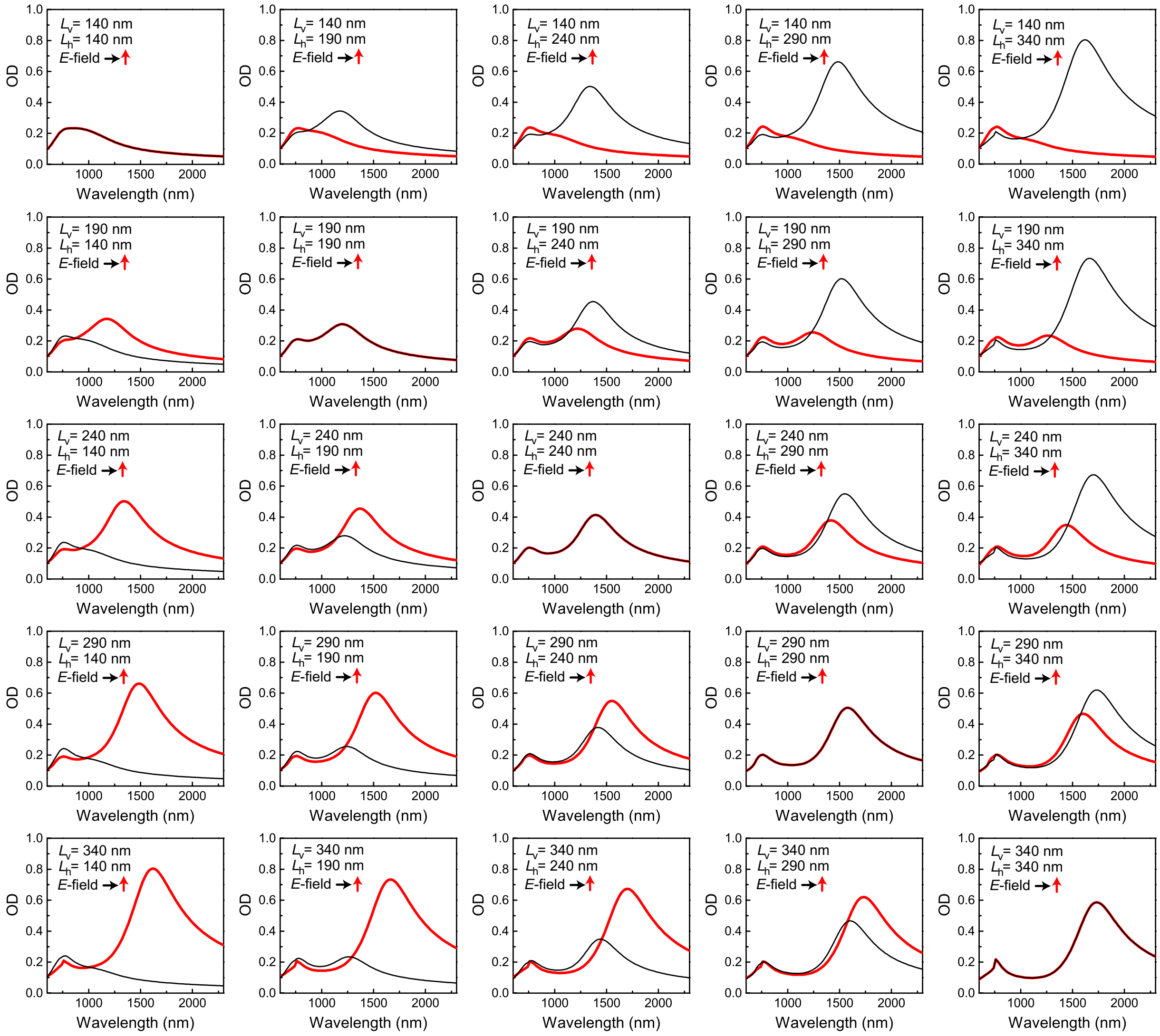

FIG. S6: Calculated extinction spectra (OD) of antenna arrays corresponding to the parameters of Figure S2, for horizontal (black curve) and vertical (red curve) polarizations.

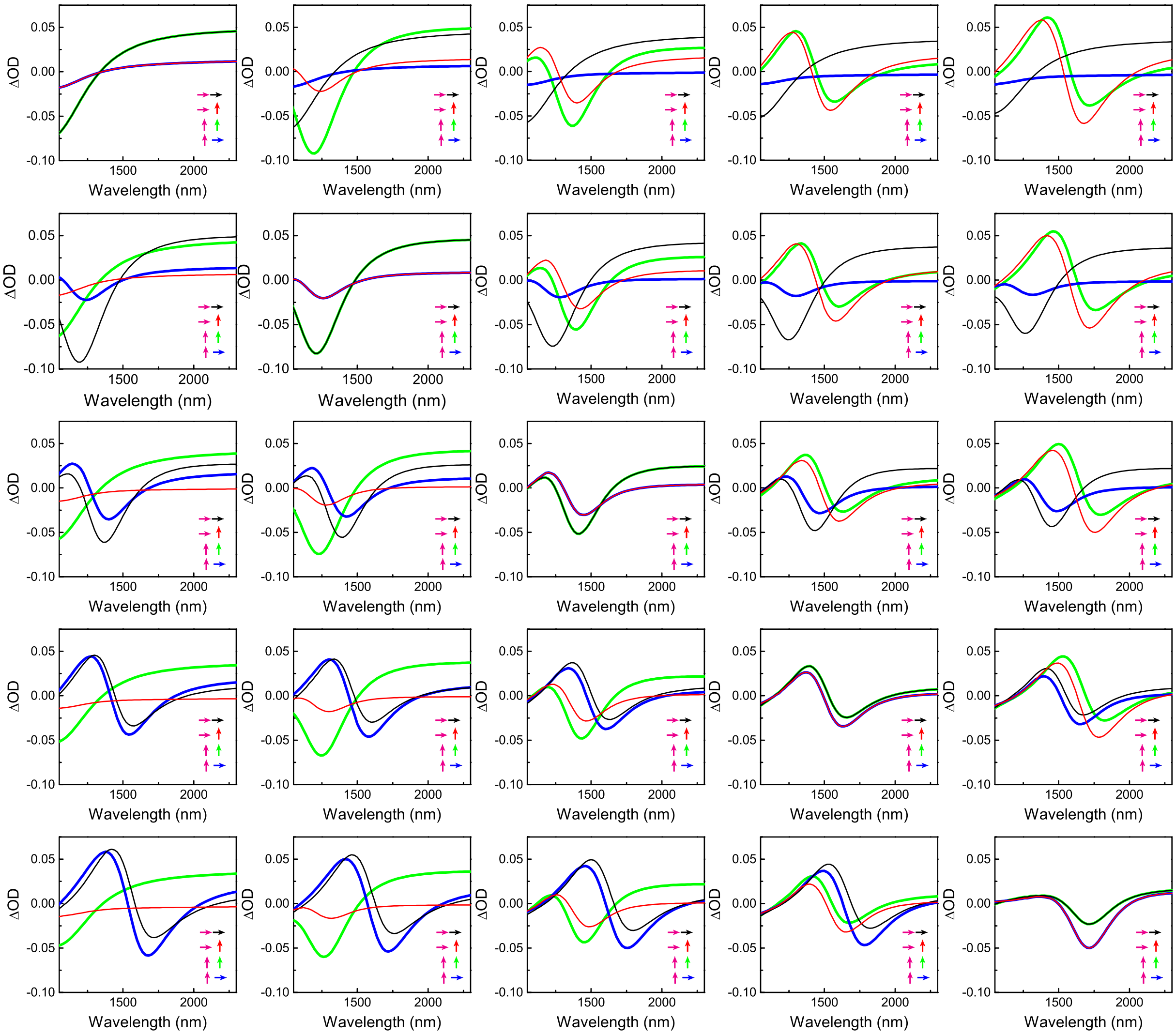

FIG. S7: Calculated differential ΔOD spectra of antenna arrays corresponding to the parameters of Figure S2.

Material	ρ g cm^{-3}	C_p J (kg K)$^{-1}$	k W(m K)$^{-1}$
Au	19.3	129	317
VO_2	4.34	690 $(T < 339\text{K} \vee T > 341\text{K})$<br>27763.5 $(339\text{K} \leq T \leq 341\text{K})$	6
FTO	7.1	363	11
SiO_2	2.203	703	1.38
air	$1.2041 \cdot 10^{-3}$	1005	0.0257

TABLE SI: Density (ρ), heat capacity at constant pressure (C_p) and thermal conductivity (k) of the various materials composing our system: gold (Au), vanadium dioxide (VO_2), silica (SiO_2) and air.

to be 1060 nm and $5 \cdot 10^7$ W cm^{-2}, respectively, according to the features of the laser used in the experiments. The second step involves the solution of the diffusion equation inside the model by means of the "Heat Transfer in Solids" COMSOL module and a "Time Domain Study". The time-dependent diffusion equation takes on the form

$$\rho C_p \frac{\partial T}{\partial t} - k\nabla^2 T = q \tag{1}$$

where ρ, C_p and k are the density, heat capacity at constant pressure and thermal conductivity of the material in each point, T is the temperature, t the independent time variable and q the heat power density at each point. The values of ρ, C_p and k for Au, VO_2, SiO_2 and air, were inferred from three seminal handbooks[5,6] and are encased in Table SI. As to q, all the absorbing points inside the system are seen as sources of heat that are active for 10 ps: this is the estimated time of illumination of the pumping-laser, after which the system is let evolve and optically recorded at the probing-time, i.e., 40 ps from the pumping switching-off (50 ps after the beginning of the pumping-probing measurement/simulation). What is actually inserted in the diffusion equations is the heat power density relative to each discretized element of the simulation domain, calculated by virtue of the formula[7]

$$q = \frac{\omega}{2}\epsilon_0 \; Im\{\epsilon\} \; |\mathbf{E}|^2 \tag{2}$$

where ω is the frequency of the pumping radiation, $\mathbf{E}$ the total electric field vector in the concerned point, ϵ_0 and ϵ the *vacuum* and local permittivity (as in all our discussion, we refer to the local or medium permittivity as the relative dimensionless permittivity). The use of the stationary solution of the electric field in this formula is justified as the optical cycle of the plasmon excitation is of the order of the femtoseconds, whereas the heat diffusion time-window investigated in this study spans over many picoseconds. Moreover, even if in principle all the (absorbing) VO_2 film can be considered as a heat source, the contributions of q more than a few hundred nanometers away from the antenna can be neglected in the picosecond time regime because of the low value of the thermal conductivity of the VO_2 (see Table SI), which shifts the significant time-scale for the diffusion in the VO_2 towards bigger values than the considered tens of picoseconds. By running the simulations we have obtained the heat power density and temperature distribution around the nanoantennas at the probing-time as depicted in Figure 3c-3e.

Finally, regarding the used mesh, tests with different mesh sizes have been performed to ensure the convergence of all the simulations: we recall that, for the nanoantennas and the VO_2 regions around them, a nanometric size of the elements composing the volume discretization were set to achieve an accurate description of the system.

C. Separating contributions from the VO_2 and antenna sources

In order to evaluate the contributions to the local phase transition from light absorption in the gold antenna and in the VO_2, we separately calculated the temperature profile by selectively removing the heat sources localized in either the substrate or the antenna, respectively. Figure S8a shows the total temperature rise caused by resonant excitation of the antenna mode of a 210 nm long gold antenna. The contributions of light absorption in the gold antenna and the VO_2 were split off in Figure S8b and c. Clearly, Figure S8b and S8c shows that absorption outside the plasmonic antenna dominates the heat source distribution under conditions of resonant pumping, and nearly completely explains the temperature increase without requiring additional energy transport from inside the nanoparticle into the VO_2 layer.

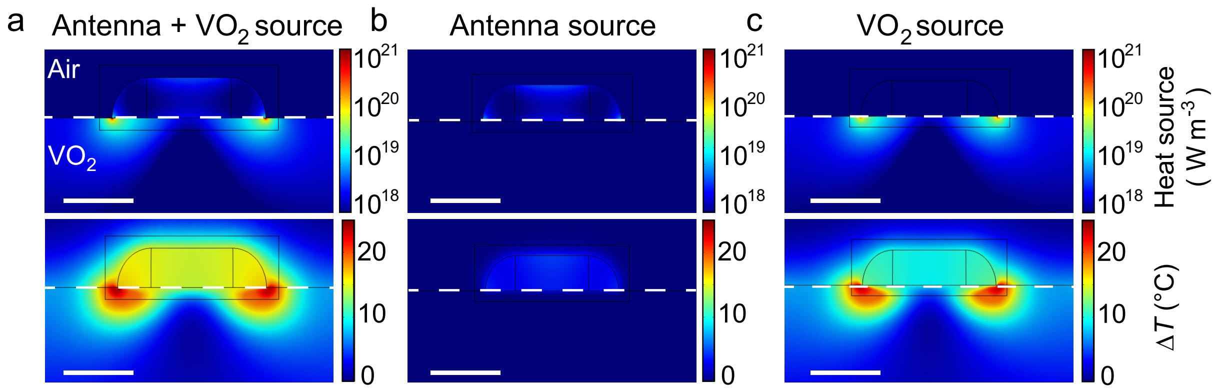

FIG. S8: (a) Heat source distribution (top panel) in antenna-VO_2 hybrid with 210 nm antenna length during the pump laser pulse, calculated using full electrodynamical model of local absorption, and temperature increase ΔT (bottom panel) calculated by using the full absorption profile (top panel) *via* time-dependent heat diffusion model. (b-c) The same as in (a) but selectively considering only the heat source contribution in either the antenna (b) or in the VO_2 substrate (c).

D. Effective IMT energy

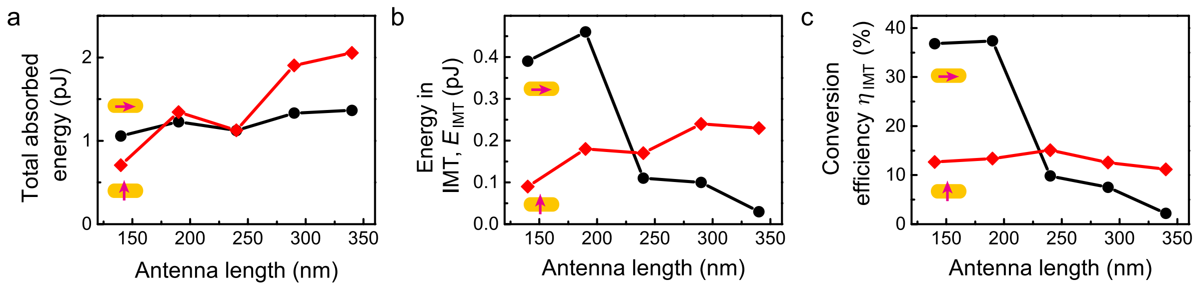

FIG. S9: Total absorbed energy (a), energy contained in phase switched volumes $E_{\rm IMT}$ (b), and resulting conversion efficiency $\eta_{\rm IMT}$ (c) of the antenna-VO_2 hybrid, for the antenna lengths under study. Arrows indicate orientations of the pump with respect to the antenna. Values are taken from the single antenna heat diffusion model as explained in the text, at a simulation time of 10 ps.

The microscopic calculations including localized absorption, heat diffusion and IMT response allow to estimate the typical energies and efficiencies involved in the process. A key figure of interest is the energy contained in the switched VO_2 regions for the various antenna geometries. We define an effective IMT energy $E_{\rm IMT}$ by summing over the volume of the phase-switched VO_2 hot-spots the contributions of the single mesh element according to the formula

$$\sum_i E_{\rm IMT} = \sum_i C_{p,i}\rho_i v_i \Delta T_i \tag{3}$$

where $C_{p,i}$, ρ_i, v_i and ΔT_i are the heat capacity (at constant pressure), density, volume and temperature increase characterizing the i-th volume element (of the discretization) residing inside the VO_2 regions undergoing IMT. We note that, in order not to miss the contributions lost *via* thermal diffusion in time, we took the ΔT_i found inside the regions with temperature greater than the critical one (i.e., T =68 °C) at the laser switching-off time, namely after 10 ps from the pumping and diffusion kick-off. The obtained values of the effective switching energy, yielded from the FEM simulations of all the 5 antenna sizes under both longitudinal and transverse polarization, are summarized in Table SII.

The energy conversion efficiency $\eta_{\rm IMT}$ is obtained by dividing the effective IMT energy $E_{\rm IMT}$ by the total energy of the simulated system (i.e., by letting run the i subscript of formula 3 over all the elements of the simulation domain) and is displayed in Table SII for all the concerned cases. Figure S9 graphically presents the data for the total energy absorbed by the antenna-VO_2 hybrid, the energy contributing to the IMT in the VO_2 hot-spots, and the overall conversion efficiency. Our calculations show a maximum conversion efficiency inside the VO_2 hot-spots up to 46%

Case	$E_{\rm IMT}$ (pJ)	$\eta_{\rm IMT}$ (a.u.)
$L = 140$ nm, longitudinal	0.39	36.8
$L = 140$ nm, transverse	0.09	12.7
$L = 190$ nm, longitudinal	0.46	37.4
$L = 190$ nm, transverse	0.18	13.4
$L = 240$ nm, longitudinal	0.11	9.8
$L = 240$ nm, transverse	0.17	15.1
$L = 290$ nm, longitudinal	0.10	7.5
$L = 290$ nm, transverse	0.24	12.6
$L = 340$ nm, longitudinal	0.03	2.2
$L = 340$ nm, transverse	0.23	11.2

TABLE SII: Effective energy in the IMT regions $E_{\rm IMT}$ and total conversion efficiency $\eta_{\rm IMT}$ of the antenna-VO_2 hybrids, for all the 5 nanoantenna lengths, and for both longitudinal and transverse polarization.

of the total absorption at resonance, i.e., for the shortest antennas when their longitudinal plasmon mode is excited by the pump laser. However this efficiency drops steeply as the antenna resonance is shifted away from the pump wavelength by increasing the antenna length. For pumping at the transverse polarization, the overall efficiency stays relatively constant within the range 12.5%-15% over the entire antenna length range.

[1] Abb M, Wang Y, de Groot CH, Muskens OL. Hotspot-mediated ultrafast nonlinear control of multifrequency plasmonic nanoantennas, *Nat Commun* 2014; **5**: 4869.

[2] Johnson PB, Christy RW. Optical constants of the noble metals, *Phys. Rev. B* 1972: **6**, 4370.

[3] Lumerical Solutions, Inc. http://www.lumerical.com/tcad-products/fdtd

[4] COMSOL Multiphysics, Inc. https://www.comsol.com/

[5] Samsonov GV. The Oxide Handbook. New York: Springer, 1973.

[6] Haynes WM. Handbook of Chemistry and Physics, 91^{st}edition. Boca Raton: CRC (2011). Palik ED. Handbook of Optical Constants of Solids, Orlando: Academic Press, 1998.

[7] Baffou G, Quidant R. Thermo-plasmonics: using metallic nanostructures as nano-sources of heat, *Laser Photon Rev* 2013; **7**: 171-187.
